# Supplementary material for: Signal categorization by foraging animals depends on ecological diversity
Source: eLife. 2019 Apr 25;8:e43965. doi: 10.7554/eLife.43965 (PMC6510532; doi:10.7554/eLife.43965)
Supplement: Supplementary file 1. — The file includes a legend to the columns of the Figure 1—source data 1 with detailed explanation of the variable codings. It is preferable to use this file as a guide to the data. [file elife-43965-supp1.pdf]

Methods Supplement eLife

Kikuchi, D. W. et al.

March 23, 2019

load packages

```
library(reshape2)
library(lattice)
library(car)

## Loading required package: carData
```

Here we read in the data.

```
mydat <- read.csv("eLifeSupplementaryData.csv")
```

KEY TO VARIABLES

combined: shape and color together to make a unique name for the prey type

treatment: treatment that subject participated in

IDno: subject's ID + filename

attacks: total number of attacks

possible: total number of prey available

color: prey color

shape: prey shape

index: in case the rows get missorted

experiment: Experiment to be interpreted as factor (Exp 1 = treatments 1-4, Exp 2 = 5-8, Exp 3 = 17-20, Exp 4 = 9-12, Exp 5 = 13-16)

unRel: unreliable trait value (zed = was not present in training)

Rel: reliable trait value (Z = was not present in training)

unRelComb: all unreliable trait values paired with same outcome in training merged into good, bad, and zed, e.g. red, purple = zed

RelComb: all reliable trait values paired with same outcome in training merged into G, B, and Z, e.g. circle, cross = good for Experiments 2-5 (Figure 1 main text).

unRelComb and **RelComb** are used to analyze the effects of traits, whereas **Rel** is used to analyze the effects of separate values of the reliable trait, e.g. circle, cross, star and triangle (Figure 1 main text).

ord: order in which a subject experienced an experiment ('order' is an R function)

Below, we put the experiments in the proper order and drop unused levels of the reliable and unreliable traits (zed and Z).

```
mydat$experiment <- as.factor(mydat$experiment)
levels(mydat$experiment) <- c('1','2','4','5','3')
mydat <- droplevels(mydat[which(mydat$unRelComb != 'zed'),])
mydat <- droplevels(mydat[which(mydat$RelComb != 'Z'),])
```

Now begins the proper description of the analysis. This RMarkdown document is to some degree redundant with Methods, but for clarity we wished to lay out both our reasoning and code in a single place.

In plain speech, we wanted to know to what degree the reliable trait (*R*) predicted subjects' decisions **relative** to the unreliable trait (*U*) in the test trial, and whether this changed between experiments. It is easier to understand what this means graphically.

Below, we visualize the raw attack rates for prey bearing different values of *R* and *U* across experiments. The "B" and "G" in the legend refer to values of *R*, and values of *U* are given along the x-axis.

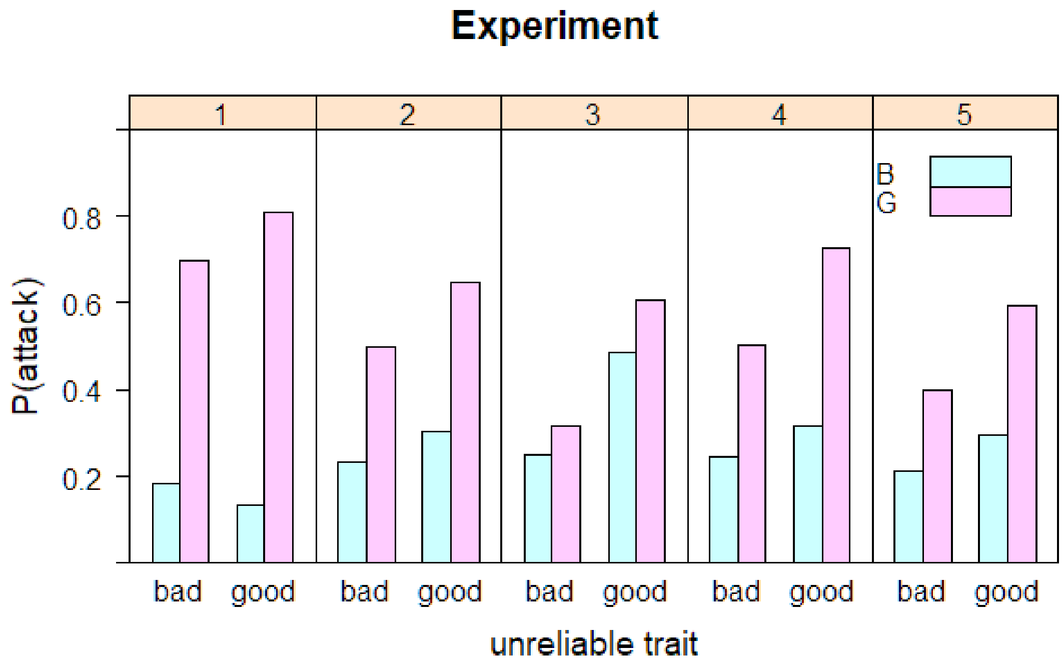

Focus on the far left panel to understand the relative importance of *R* and *U* in just Experiment 1. The blue bars (short) are the probability of attacking prey with values of *R* that were associated with 'bad' prey during training. The pink bars (tall) are the probability

of attacking prey with values of *R* that were associated 'good' prey during training. The average difference between the blue and pink bars is the effect of *R*. Now look at the x-axis: there are two labels, good and bad. The average difference between the bars above the good and bad labels is the effect of *U*. To find the relative importance of *R* versus *U* in Experiment 1, we needed to find the difference in the their effects in that experiment.

Once we did this for each experiment, we needed to compare the relative importance of *R* versus *U* **across** experiments, to see if it changed significantly with species richness or evenness.

In other words, we needed to find the difference between the effects of *R* and *U* in each experiment, and then find the difference of those differences in a pairwise fashion between experiments.

There was not a simple model that we could use to do this analysis, but we could use a model to give us both the estimates for the effects of *R* and *U*, and their standard errors. Then we used additional functions to calculate the differences that we needed (first, differences between *R* and *U* within each experiment, and second, pairwise differences of those difference among experiments), and estimates of uncertainty in those differences.

The model we needed to build had to include *R*, *U*, and experiment as factors. Each subject also participated in multiple experiments. Although the order of experiments was randomized, to account for any influence it might have had on the estimates we were making, we included interactions with it as well. Although we were not interested in order, we included it as an interaction term lest it influence estimates of the effects of *R* and *U*. By centering order and our other predictors, we could still interpret the coefficients we were interested in, even if significant interactions with order existed (Schielzeth 2010).

This code centers order

```
mydat$ordC <- scale(mydat$ord, center = T, scale = F)
```

We also used the contrasts argument to center the reliable and unreliable traits directly in the call to the model function.

Below is the model that we used to extract the effects of *R* and *U* and their standard errors. Normally, one would include *R* and *U* as main effects (and an intercept!), but Schielzeth (2010) points out that without them, the estimates for each group are given in the model output directly. We were not interested in using this model to test hypotheses, but rather as a tool to extract means, so violating marginality here was not the problem it would usually be. Experiment was included as a main effect, because the mean for each experiment was the reference level against which the effect sizes of *R* and *U* are calculated.

We used logistic regression, because the response variable was binary. This was advantageous for finding the differences in effect sizes because the output units would be in log odds. Finding the difference of two log odds indicated how large the effect of one variable was over another. For example, a difference of 1 in log odds between *R* and *U* would indicate that *R* has exp(1) = 2.72 times the importance to a subject's decisions than *U*. See main text for more explanation.

Furthermore, we included subject IDno as a fixed effect, because multiple observations were taken from each subject. Normally, one would simply use a glmm with IDno as a random effect, but those models failed to converge.

```
m.3way <- glm(cbind(attacks,possible-attacks) ~
                0 + experiment + RelComb:experiment + unRelComb:experiment +
                RelComb:experiment:ordC + unRelComb:experiment:ordC + IDno,
              contrasts = list(experiment = matrix(c(-.2,-.2,-.2,-.2,
                                                     .8,-.2,-.2,-.2,
                                                     -.2,.8,-.2,-.2,
                                                     -.2,-.2,.8,-.2,
                                                     -.2,-.2,-.2,.8),
                                                   5,4, byrow = T),
                               unRelComb = matrix(c(-0.5,0.5),2,1),
                               RelComb = matrix(c(-0.5,0.5),2,1)),
              data = mydat, family = 'binomial')
```

What does the output of the model we fit actually look like, and how does one interpret it?

```
round(coef(summary(m.3way)),3)[c(1:5,50:59),]

##                         Estimate Std. Error z value Pr(>|z|)
## experiment1               -0.948      0.265  -3.573    0.000
## experiment2               -0.968      0.245  -3.950    0.000
## experiment4               -0.784      0.245  -3.204    0.001
## experiment5               -1.130      0.245  -4.609    0.000
## experiment3               -1.041      0.245  -4.245    0.000
## experiment1:RelComb1       3.357      0.257  13.062    0.000
## experiment2:RelComb1       1.516      0.144  10.562    0.000
## experiment4:RelComb1       1.662      0.145  11.431    0.000
## experiment5:RelComb1       1.212      0.143   8.487    0.000
## experiment3:RelComb1       0.401      0.148   2.715    0.007
## experiment1:unRelComb1     0.237      0.239   0.992    0.321
## experiment2:unRelComb1     0.585      0.142   4.133    0.000
## experiment4:unRelComb1     0.762      0.144   5.307    0.000
## experiment5:unRelComb1     0.717      0.141   5.076    0.000
## experiment3:unRelComb1     1.371      0.150   9.141    0.000
```

The first five terms are estimates for the rates of attack by subjects in each experiment. They themselves are not interesting. Obviously, we were not interested in the next 44 terms either - they are the individual effects we controlled for.

We were interested in the next five terms. They give - for each experiment - the difference in attack rate on prey with values of R paired with 'good' prey and values of R paired with 'bad' prey. In other words, they give the effect of R in each experiment and its standard error. Likewise, the five terms following those give the effect of U in each experiment, and its standard error. The Wald z- and p-values for experiment test the hypotheses that estimates for each experiment differ from zero: not very interesting. But the z- and p-values for experiment:R and experiment:U test the hypotheses that R and U had significant effects

on subjects' decisions in each experiment. This was interesting. They always did, except for *U* in Experiment 1, where it was completely outcompeted by *R*.

The remaining terms describe the interactions between order, *U*, *R*, and experiment. They were not interesting for testing the predictions that are made by the hypotheses in this manuscript.

We wanted to find how *R* - *U* changed across experiments. In other words, we wanedt to find *R* - *U* for each experiment, and then see if those estimates fell into different groups for each experiment.

In finding *R* - *U*, we calculated a function of effects (parameters) estimated by the model. It seems straightforward to do this - one could simply subtract one effect from the other - but finding the standard errors of the new term can be tricky, especially with unbalanced designs like this one. One way to approximate the standard errors of functions of parameters is the delta method, which finds the new variance as a function of the derivatives of the two original parameters (Bolker 2007). The deltaMethods function in the car package can do this, but requires text strings to describe the functions being calculated. Characters such as ":" that are describe interactions in R will not work, so we needed to create new parameter names for the deltaMethod function.

```
pnames <- c('e1','e2','e4','e5','e3',1:44,
            'r1','r2','r4','r5','r3', #we care about these
            'u1','u2','u4','u5','u3', #and these
            60:74)
```

Next, we calculated the differences between *R* and *U* for each experiment, with 95% confidence intervals for display. This was the first difference calculation.

```
D1 <- rbind(deltaMethod(m.3way, 'r1 - u1',
                        level = 0.95, parameterNames = pnames),
            deltaMethod(m.3way, 'r2 - u2',
                        level = 0.95, parameterNames = pnames),
            deltaMethod(m.3way, 'r3 - u3',
                        level = 0.95, parameterNames = pnames),
            deltaMethod(m.3way, 'r4 - u4',
                        level = 0.95, parameterNames = pnames),
            deltaMethod(m.3way, 'r5 - u5',
                        level = 0.95, parameterNames = pnames))
colnames(D1) <- c('estimate','SE','lower95','upper95')
```

But this didn't yet tell us if the new terms we calculated ought to be placed in different groups. To find this out, we needed to perform the second difference calculation, which was pairwise for *R* - *U* between experiments.

This was easy enough with the delta method, as we simply had to use a more elaborate function, e.g. *(R.E1-U.E1) - (R.E2-U.E2)*. If the confidence interval of this difference overlaps with 0 for a comparison, it means there was no statistically significant difference between the reliable and unreliable traits between the two experiments.

Multiple pairwise comparisons increase the probability of Type I error, so we used the Bonferroni correction to adjust the p-value associated with significance. We did not actually calculate the p-value of each pairwise comparison, however, since we consider estimates and confidence intervals more meaningful.

```
D2 <- rbind(deltaMethod(m.3way, '(r1 - u1) - (r2 - u2)',
                        level = 0.995, parameterNames = pnames),
            deltaMethod(m.3way, '(r1 - u1) - (r3 - u3)',
                        level = 0.995, parameterNames = pnames),
            deltaMethod(m.3way, '(r1 - u1) - (r4 - u4)',
                        level = 0.995, parameterNames = pnames),
            deltaMethod(m.3way, '(r1 - u1) - (r5 - u5)',
                        level = 0.995, parameterNames = pnames),
            deltaMethod(m.3way, '(r2 - u2) - (r3 - u3)',
                        level = 0.995, parameterNames = pnames),
            deltaMethod(m.3way, '(r2 - u2) - (r4 - u4)',
                        level = 0.995, parameterNames = pnames),
            deltaMethod(m.3way, '(r2 - u2) - (r5 - u5)',
                        level = 0.995, parameterNames = pnames),
            deltaMethod(m.3way, '(r3 - u3) - (r4 - u4)',
                        level = 0.995, parameterNames = pnames),
            deltaMethod(m.3way, '(r3 - u3) - (r5 - u5)',
                        level = 0.995, parameterNames = pnames),
            deltaMethod(m.3way, '(r4 - u4) - (r5 - u5)',
                        level = 0.995, parameterNames = pnames))
round(D2, 4)
```

```
##                       Estimate     SE  0.25 % 99.75 %
## (r1 - u1) - (r2 - u2)   2.1892 0.3896  1.0956  3.2828
## (r1 - u1) - (r3 - u3)   4.0898 0.3976  2.9737  5.2058
## (r1 - u1) - (r4 - u4)   2.2196 0.3887  1.1286  3.3107
## (r1 - u1) - (r5 - u5)   2.6256 0.3897  1.5318  3.7195
## (r2 - u2) - (r3 - u3)   1.9006 0.2829  1.1063  2.6948
## (r2 - u2) - (r4 - u4)   0.0304 0.2711 -0.7307  0.7915
## (r2 - u2) - (r5 - u5)   0.4364 0.2723 -0.3281  1.2009
## (r3 - u3) - (r4 - u4)  -1.8701 0.2816 -2.6607 -1.0796
## (r3 - u3) - (r5 - u5)  -1.4641 0.2826 -2.2573 -0.6710
## (r4 - u4) - (r5 - u5)   0.4060 0.2710 -0.3547  1.1667
```

Above, we can see that *R* - *U* in the first experiment was significantly different from all others, and that *R* - *U* in the third experiment was also different from all others, but that Experiments 2, 4, and 5 all belonged to the same group.

The results are plotted below.

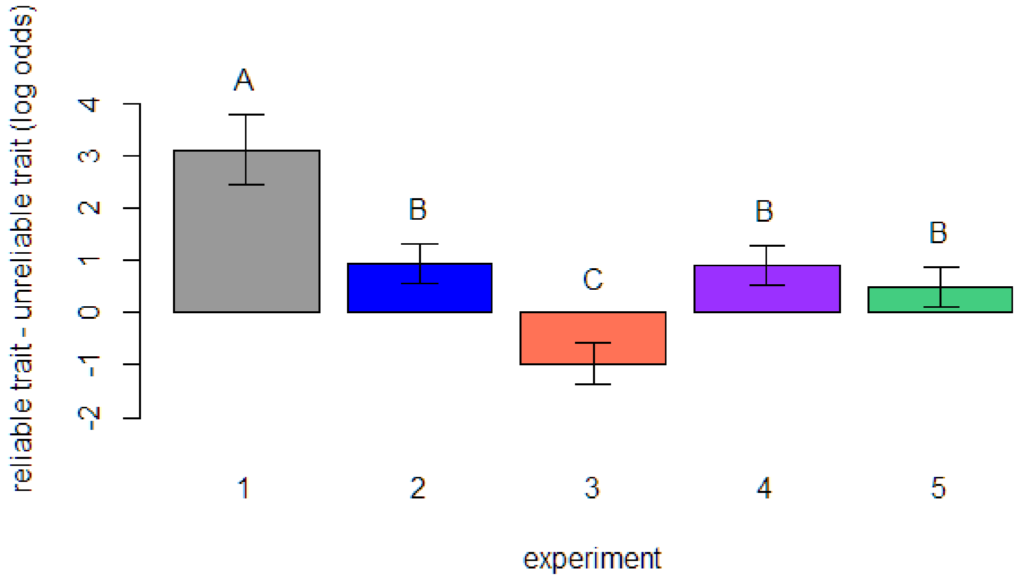

It was also necessary to know how much subjects discriminated in total in each experiment, so that there is some context to interpret the relative importance of the reliable and unreliable traits: if subjects had hardly discriminated at all between prey, they might as well have been guessing randomly. To test this, we calculated $R + U$ in each experiment.

```
T1 <- rbind(deltaMethod(m.3way, 'r1 + u1',
                        level = 0.95, parameterNames = pnames),
            deltaMethod(m.3way, 'r2 + u2',
                        level = 0.95, parameterNames = pnames),
            deltaMethod(m.3way, 'r3 + u3',
                        level = 0.95, parameterNames = pnames),
            deltaMethod(m.3way, 'r4 + u4',
                        level = 0.95, parameterNames = pnames),
            deltaMethod(m.3way, 'r5 + u5',
                        level = 0.95, parameterNames = pnames))
colnames(T1) <- c('estimate','SE','lower95','upper95')
```

Again, pairwise comparisons with Bonferroni correction were applied to determine if this differed significantly among experiments.

```
T2 <- rbind(deltaMethod(m.3way, '(r1 + u1) - (r2 + u2)',
                        level = 0.995, parameterNames = pnames),
            deltaMethod(m.3way, '(r1 + u1) - (r3 + u3)',
                        level = 0.995, parameterNames = pnames),
            deltaMethod(m.3way, '(r1 + u1) - (r4 + u4)',
                        level = 0.995, parameterNames = pnames),
            deltaMethod(m.3way, '(r1 + u1) - (r5 + u5)',
                        level = 0.995, parameterNames = pnames),
```

```
             deltaMethod(m.3way, '(r2 + u2) - (r3 + u3)',
                         level = 0.995, parameterNames = pnames),
             deltaMethod(m.3way, '(r2 + u2) - (r4 + u4)',
                         level = 0.995, parameterNames = pnames),
             deltaMethod(m.3way, '(r2 + u2) - (r5 + u5)',
                         level = 0.995, parameterNames = pnames),
             deltaMethod(m.3way, '(r3 + u3) - (r4 + u4)',
                         level = 0.995, parameterNames = pnames),
             deltaMethod(m.3way, '(r3 + u3) - (r5 + u5)',
                         level = 0.995, parameterNames = pnames),
             deltaMethod(m.3way, '(r4 + u4) - (r5 + u5)',
                         level = 0.995, parameterNames = pnames))
round(T2,4)

##                          Estimate     SE  0.25 % 99.75 %
## (r1 + u1) - (r2 + u2)    1.4932 0.4181  0.3197  2.6668
## (r1 + u1) - (r3 + u3)    1.8231 0.4203  0.6434  3.0028
## (r1 + u1) - (r4 + u4)    1.1709 0.4214 -0.0121  2.3539
## (r1 + u1) - (r5 + u5)    1.6653 0.4178  0.4924  2.8382
## (r2 + u2) - (r3 + u3)    0.3298 0.2990 -0.5096  1.1692
## (r2 + u2) - (r4 + u4)   -0.3223 0.3008 -1.1668  0.5221
## (r2 + u2) - (r5 + u5)    0.1721 0.2955 -0.6575  1.0016
## (r3 + u3) - (r4 + u4)   -0.6522 0.3037 -1.5048  0.2005
## (r3 + u3) - (r5 + u5)   -0.1578 0.2985 -0.9957  0.6802
## (r4 + u4) - (r5 + u5)    0.4944 0.3003 -0.3487  1.3375
```

Results below show that although subjects discriminated more in Experiment 1, generally they discriminated in all experiments.

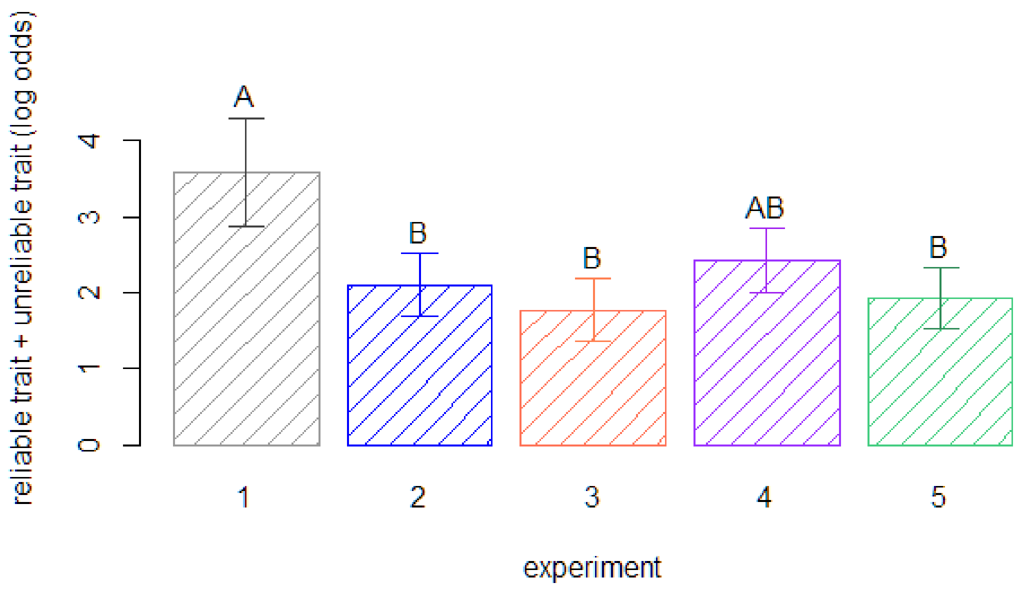

There remained one final analysis to do. In the rich uneven community (Experiment 5), subjects used *R* relatively more than in the rich even community of Experiment 3. In fact, they used *R* about as much as subjects in Experiment 2 and Experiment 4 did. It may be that in uneven communities, subjects focused on the most abundant good prey, ignoring other types of prey. Reliance on this most abundant "alternative" prey species makes generalization using *U* less likely, while also making other values of *R* less important. If this hypothesis were true, then subjects should have focused more on the most abundant values of *R* in Experiments 4 and 5.

We tested this by looking at distinct values of *R*, rather than simply lumping them together into "G" and "B" values. The variable **Rel** does this, containing two values of good prey, and two values of bad prey. "G1" denotes the most abundant good prey in uneven communities.

```
valmelt <- melt(tapply(mydat$attacks,list(mydat$unRel,mydat$Rel,
                                           mydat$experiment),sum))
colnames(valmelt) <- c('unRel','Rel','experiment','attacks')
pvalmelt <- melt(tapply(mydat$possible,list(mydat$unRel,mydat$Rel,
                                             mydat$experiment),sum))
valmelt$p.attacks <- valmelt$attacks/pvalmelt[,4]
```

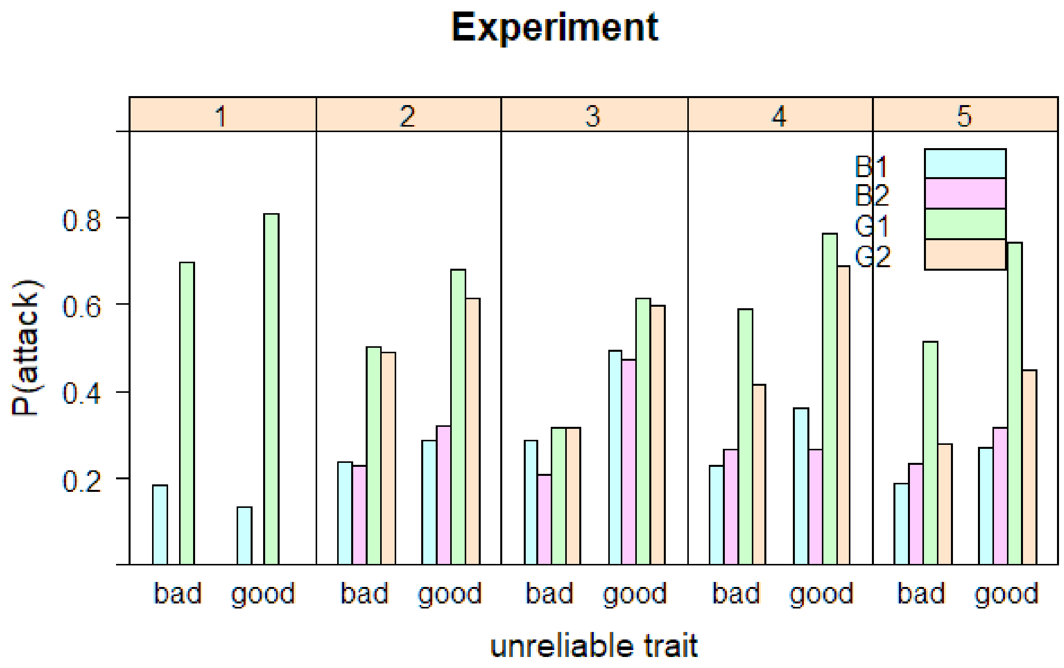

It seems obvious from the figure above that subjects do indeed tend to learn to strongly prefer 'G1' prey over all others in Experiment 5, and to some degree in Experiment 4, yet to test this is so statistically, we had to test whether or not there was a significant improvement in model fit by using the different values of prey in **Rel** rather than the simple "good" and "bad" values of **RelComb**. This was straightforward to do with the likelihood ratio test:

```
m.val <- glm(cbind(attacks,possible-attacks) ~
              0 + experiment + unRelComb:experiment + Rel:experiment +
```

```
              unRelComb:Rel:experiment +
              unRelComb:experiment:ordC + Rel:experiment:ordC + IDno,
              contrasts = list(experiment = matrix(c(-.2,-.2,-.2,-.2,
                                                     .8,-.2,-.2,-.2,
                                                     -.2,.8,-.2,-.2,
                                                     -.2,-.2,.8,-.2,
                                                     -.2,-.2,-.2,.8),
                                                   5,4, byrow = T),
                               unRelComb = matrix(c(-0.5,0.5),2,1),
                               Rel = matrix(c(-.25,-.25,-.25,
                                              .75,-.25,-.25,
                                              -.25,.75,-.25,
                                              -.25,-.25,.75),
                                            4,3, byrow = T)),
              data = mydat, family = 'binomial')

anova(m.3way, m.val, test = "Chisq")

## Analysis of Deviance Table
##
## Model 1: cbind(attacks, possible - attacks) ~ 0 + experiment +
RelComb:experiment +
##     unRelComb:experiment + RelComb:experiment:ordC +
unRelComb:experiment:ordC +
##     IDno
## Model 2: cbind(attacks, possible - attacks) ~ 0 + experiment +
unRelComb:experiment +
##     Rel:experiment + unRelComb:Rel:experiment + unRelComb:experiment:ordC
+
##     Rel:experiment:ordC + IDno
##   Resid. Df Resid. Dev Df Deviance  Pr(>Chi)
## 1      1514     4507.1
## 2      1485     4420.8 29   86.328 1.323e-07 ***
## ---
## Signif. codes:  0 '***' 0.001 '**' 0.01 '*' 0.05 '.' 0.1 ' ' 1
```

The difference in model fit was large, and highly significant. Clearly, the more finely categorized values of *R* described in **Rel** contained useful information not preserved in the coarser categories described in **RelComb**: subjects prefered ‘G1’ to other values of the reliable trait, even ‘G2’.
